# p21-Activated Kinase 1 Is Permissive for the Skeletal Muscle Hypertrophy Induced by Myostatin Inhibition

**DOI:** 10.3389/fphys.2021.677746

**Published:** 2021-06-17

**Authors:** Caroline Barbé, Audrey Loumaye, Pascale Lause, Olli Ritvos, Jean-Paul Thissen

**Affiliations:** ^1^Pole of Endocrinology, Diabetes and Nutrition, Institute of Clinical and Experimental Research, Catholic University of Louvain, Brussels, Belgium; ^2^Department of Physiology, Faculty of Medicine, University of Helsinki, Helsinki, Finland

**Keywords:** myostatin, follistatin, sActRIIB, PAK1, skeletal muscle hypertrophy

## Abstract

Skeletal muscle, the most abundant tissue in the body, plays vital roles in locomotion and metabolism. Understanding the cellular processes that govern regulation of muscle mass and function represents an essential step in the development of therapeutic strategies for muscular disorders. Myostatin, a member of the TGF-β family, has been identified as a negative regulator of muscle development. Indeed, its inhibition induces an extensive skeletal muscle hypertrophy requiring the activation of Smad 1/5/8 and the Insulin/IGF-I signaling pathway, but whether other molecular mechanisms are involved in this process remains to be determined. Using transcriptomic data from various Myostatin inhibition models, we identified *Pak1* as a potential mediator of Myostatin action on skeletal muscle mass. Our results show that muscle PAK1 levels are systematically increased in response to Myostatin inhibition, parallel to skeletal muscle mass, regardless of the Myostatin inhibition model. Using *Pak1* knockout mice, we investigated the role of *Pak1* in the skeletal muscle hypertrophy induced by different approaches of Myostatin inhibition. Our findings show that *Pak1* deletion does not impede the skeletal muscle hypertrophy magnitude in response to Myostatin inhibition. Therefore, *Pak1* is permissive for the skeletal muscle mass increase caused by Myostatin inhibition.

## Introduction

Myostatin (MSTN), a member of the TGF-β family primarily expressed in skeletal muscle, is a negative regulator of skeletal muscle mass, as shown by the marked increase in skeletal muscle mass following MSTN inhibition or gene deletion (Mcpherron et al., 1997; Grobet et al., 2003). Several approaches may be used to inhibit MSTN. Among these, the physiological MSTN binding protein, Follistatin (FS), and the soluble Activin IIB receptor (sActRIIB), have been shown to be promising therapeutic strategies for reversing muscle wasting (Busquets et al., 2012) and dystrophies (Rodino-Klapac et al., 2013). Previous works from us and others have demonstrated that muscle fiber hypertrophy induced by MSTN inhibition results from increased protein synthesis and requires the activation of Smad 1/5/8 (Winbanks et al., 2013) and the Insulin/IGF-I pathway (Kalista et al., 2012; Winbanks et al., 2012; Barbé et al., 2015). However, the molecular mechanisms responsible for the muscle phenotype induced by MSTN inhibition remain not fully understood.

The p21-activated kinase 1 (PAK1) is a 68-kDa Serine/Threonine kinase of the PAK1–6 family, which plays a role in many cellular processes as evidenced by its more than 40 substrates identified to date. PAK proteins are divided into two groups, with PAK1–3 belonging to group 1 and PAK 4–6 belonging to group 2 (Bokoch, 2003). From Group 1 PAKs, only PAK1 and PAK2 have been detected in skeletal muscle (Tunduguru et al., 2014; Joseph et al., 2017). While *Pak1* or *Pak2* deletion does not affect muscle mass, mice lacking both *Pak1* and *Pak2* display reduced muscle mass indicating a potential redundancy of these two proteins (Joseph et al., 2017). Recent evidences have highlighted the role of *Pak1* in muscle mass regulation (Martinelli et al., 2016; Joseph et al., 2017; Cerquone Perpetuini et al., 2018), but its implication in muscle hypertrophy caused by MSTN inhibition has never been explored to date. In a first step, we identified *Pak1* as the only gene consistently upregulated in skeletal muscle following various MSTN inhibition models. Given the anti-atrophic role of *Pak1* in skeletal muscle (Cerquone Perpetuini et al., 2018) and its upregulation in response to MSTN inhibition, we hypothesized that increased *Pak1* could mediate the muscle mass increase induced by MSTN inhibition. Then, we explored the role of *Pak1* in both the magnitude and the onset of skeletal muscle hypertrophy following MSTN inhibition. Our findings show that *Pak1* deletion did not affect the magnitude of muscle hypertrophy caused by MSTN inhibition.

## Materials and Methods

### Animals

All the mouse experiments were performed with the approval of the Committee for Ethical Practices in Animal Experiments of the Catholic University of Louvain (Brussels, Belgium). Male mice were housed under standardized conditions of light (12:12 h light–dark cycle) and temperature (22 ± 2°C) with free access to standard chow pellets and water. Animals were euthanized by decapitation after CO_2_ administration and tibialis anterior (TA), gastrocnemius (GC), soleus (SOL), and extensor digitorum longus (EDL) muscles were collected for further analysis. For a complete overview of the different animal models including the age at the start of the experiment, see Table 1.

**TABLE 1:** Overview of the different animal models.

Models	Age	Description	Phenotype
MSTN KO	8 weeks	FVB mice harboring a constitutive deletion of the third MSTN exon	Hypertrophy
mTrFS	6 weeks	C57Bl/6 transgenic mice overexpressing the human FS 288 specifically in skeletal muscle	Hypertrophy
pM1-hFS288	14 weeks	Plasmid coding for the human FS 288 fused to the c-myc tag was transfected into TA muscle of C57Bl/6 mice which were euthanized 17 days later	Hypertrophy
sActRIIB 1x	8 weeks	C57Bl/6 mice treated with 1 injection of 10 mg/kg sActRIIB or PBS and euthanized 48 h after the injection	Hypertrophy
sActRIIB 2x	8–9 weeks	C57Bl/6 mice treated with 2 injections of 10 mg/kg sActRIIB or PBS, with injections being 48 h apart, and euthanized 48 h after the last injection	Hypertrophy
sActRIIB 4x	8–9 weeks	C57Bl/6 mice treated with 4 injections of 10 mg/kg sActRIIB or PBS, with injections being 48 to 72 h apart, and euthanized 48 h after the last injection	Hypertrophy
pcDNA-MSTN	8 weeks	Plasmid coding for the murine MSTN transfected into TA muscle of FVB mice which were harvested 14 days later	Atrophy
DEXA	8 weeks	FVB mice treated by a daily subcutaneous injection of 5 mg/kg DEXA for 4 days	Atrophy
*Pak1* KO	8–9 weeks	C57Bl/6 mice presenting a whole-body 2 kb deletion of the genomic *Pak1* DNA	None

#### Models of Muscle Hypertrophy

Different animal models of MSTN inhibition were used in this work: FVB MSTN knockout (KO) mice and their control wild-type (WT) littermates (Grobet et al., 2003); C57Bl/6 transgenic (mTrFS) mice and their control WT littermates (Lee and Mcpherron, 2001); C57Bl/6 mice transfected with human FS gene (pM1-hFS288) (Barbé et al., 2015); and C57Bl/6 mice treated by intraperitoneal (ip) injections with sActRIIB (Hulmi et al., 2013). The sActRIIB is a soluble ligand-binding domain of Activin receptor type IIB fused to Fc domain IgG that causes MSTN and Activin A inhibition (Hoogaars et al., 2012).

#### Models of Muscle Atrophy

FVB mice transfected with murine MSTN gene (pcDNA-MSTN) (Durieux et al., 2007) and FVB mice subcutaneously injected with dexamethasone (DEXA, Aacidexam^®^; Organon BioSciences) (Gueugneau et al., 2018) were used.

#### *Pak1* Knockout Mice

To investigate the role of *Pak1* in muscle hypertrophy induced by MSTN inhibition, C57Bl/6 *Pak1* KO mice and their control WT littermates were used (Allen et al., 2009).

### mRNA Analysis by Real-Time Quantitative (RTQ)-PCR

Total RNA was isolated from the frozen TA and GC muscles using TRIzol reagent^®^ as described by the manufacturer. Recovery was 1 μg/mg of muscle. Reverse transcription and real-time quantitative PCR were done as previously described (Dehoux et al., 2004). Primers were tested in order to avoid primers dimers, self-priming formation or unspecific amplifications and Glyceraldehyde-3-Phosphate Dehydrogenase (*Gapdh*) was used as reporter gene. Primers were designed to have standardized optimal PCR conditions. Accession numbers for the sequences and primers used were: *Pak1*: NM_011035.2 (TCCGCCAGATGCTTTGACCCG-AATGG CCACCTCCTGCCCTGT), *Pak2*: NM_177326.3 (AGCACCGG AGGAAAAGATCC-TGCCAGTGAACTCTCCCGTA), and *Gapdh*: AF106860 (TGCACCACCAACTGCTTA-GGATGCA GGGATGATGTTC). We confirmed that the expression values of GAPDH normalized to RNA were not affected by MSTN inhibition in our different animal models. This was confirmed at the protein level, showing that the abundance of GAPDH protein was not affected by FS overexpression, *Mstn* deletion, sActRIIB treatment, DEXA treatment and *Pak1* deletion, making GAPDH a valuable control.

### Western Blot Analysis

Frozen GC muscles, previously pestled in liquid nitrogen, were homogenized (100 mg/ml) with Ultraturrax (IKA Labortechnik) in ice-cold pH 7.0 buffer containing 20 mM Tris, 270 mM sucrose, 5 mM EGTA, 1 mM EDTA, 1 mM sodium orthovanadate, 50 mM β-glycerophosphate, 5 mM sodium pyrophosphate, 50 mM sodium fluoride, 1 mM DTT (1,4-dithiothreitol), 1% (v/v) Triton X-100, and 10% protease inhibitor cocktail (Roche Applied Science). Homogenates were centrifuged at 10,000 × *g* for 10 min at 4°C and supernatants were immediately stored at −80°C. Equal amounts of proteins were resolved by sodium dodecyl sulfate-polyacrylamide gel electrophoresis and transferred to polyvinylidene fluoride membranes. Membranes were incubated overnight at 4°C in TBST containing 1% bovine serum albumin with the following primary antibodies: anti-phospho-Smad3 Ser^423/425^ (Cell signaling #9520) and anti-Smad3 (Cell signaling #9523) at 1:250 dilution; anti-phospho-AKT Ser^473^ (Cell signaling #9271), anti-AKT (Cell signaling #9272), anti-phospho-4E-BP1 Ser^65^ (Cell signaling #9451), anti-4E-BP1 (Cell signaling #9452), anti-phospho-P70S6K Thr^389^ (Cell signaling #9205), anti-phospho-FOXO3a Ser^253^ (Cell signaling #9466), anti-FOXO3a (Cell signaling #2497), and anti-PAK2 (Cell signaling #2615) at 1:500 dilution; anti-S6 (Cell signaling #2217) and anti-PAK1 (Cell signaling #2602) at 1:1,000 dilution; anti-phospho-S6 (Cell signaling #4858) at 1:2,000 dilution. Then membranes were incubated with a goat anti-rabbit horseradish peroxidase (HRP)-conjugated secondary antibody (Cell Signaling #7074) at 1:2,000 dilution for 1 h and developed using Enhanced Chemiluminescence (ECL) Western blotting Detection System Plus (GE Healthcare). Developed films were scanned and signal quantification was determined using Image J software as described before (Gueugneau et al., 2018). Ponceau Red staining was used as a loading control (Moritz, 2017).

### Expression Plasmids and DNA Preparation

The pcDNA-MSTN plasmid coding for the murine MSTN was kindly provided by D. Freyssenet (Saint Étienne, France) (Durieux et al., 2007). The pM1-hFS288 c-myc plasmid coding for the human FS containing 288 amino acids was constructed as previously described (Gilson et al., 2009; Kalista et al., 2012). Empty plasmids (pcDNA and pM1) were used as control plasmids. Plasmids were amplified in *Escherichia coli* top 10 F’ (Invitrogen) and purified with an EndoFree plasmid giga kit (QIAGEN). Plasmids were stored at −80°C. The day before injection, 30 μg of plasmid were lyophilized and resuspended in 30 μl 0.9% NaCl solution.

### DNA Electrotransfer

Each animal was anesthetized with a mixture of 100 mg/kg ketamine (Anesketin^®^; Pfizer) and 15 mg/kg xylazine hydrochloride (Rompun^®^; Bayer) administered by ip injection. Thirty microliters of plasmid solution (1 μg/μl) were injected into each TA muscle using a Hamilton syringe with a 30-gauge needle (pcDNA or pM1 in the right TA and pcDNA-MSTN or pM1-hFS288 in the left TA), and the muscles were then electroporated using the electroporation conditions described by Bloquel et al. (2004) (8 pulses of 200 V/cm and 20 ms per pulse at 2 Hz). The mice were euthanized 14 or 17 days after electroporation of pcDNA-MSTN or pM1-hFS288, respectively.

### Histological Analysis

Tibialis anterior muscles were dissected and immediately fixed with buffered formalin for 48 h and embedded in paraffin for histological analysis. Serial sections (5 μm thick) were cut and mounted on glass slides (Superfrost Plus; Menzel-Glaser). Muscle sections were deparaffinized and blocked in PBS-BSA (5%) containing normal goat serum (4%) for 30 min at RT. To evaluate the effect of FS on muscle fibers cross-sectional area (CSA), FS-c-myc was detected by immunohistochemistry with a rabbit polyclonal anti-c-myc (1:800; Bethyl Laboratories) for 1 h at RT. Primary antibody was detected by applying for 30 min at RT a biotinylated second antibody, which was a goat anti-rabbit conjugated to peroxidase-labeled polymer (Dako). Peroxidase activity was revealed with DAB substrate (Chemicon International), which produces a brown stain. The sections were counterstained with Mayer’s hematoxylin, rinsed, and mounted in Faramount (Dako). Muscle fiber CSA were measured with a microscope Axio-Star (Carl Zeiss) coupled to a Zeiss Axiocam digital camera MRc and to image analyzer software (Axiovision software version 4.7; Carl Zeiss). All the positive muscle fibers in the FS-transfected TA muscles were measured (18 ± 3 in *Pak1* KO mice vs. 69 ± 4 in WT mice). Two hundred negative fibers, randomly chosen in the contralateral TA transfected with insertless plasmid pM1, were measured and considered as controls.

### Muscle Strength and Endurance Test Protocol

Maximal muscle force was determined by a grip test of both forelimb and combined fore- and hindlimb muscles. Limb strength was recorded using a grid connected to a sensor (Bioseb - Panlab). The mice were gently laid on the top of the grid so that their front paws (forelimb test) or both their four paws (fore and hindlimb test) can grip the grid. Then mice were pulled back steadily until the grip was released down the complete length of the grid. Two series of three tests were performed at an interval of 15 min. Results are presented as the highest value of force recorded, related to body weight. Endurance tolerance was determined by an incremental treadmill exercise test. The test was started at low velocity (5 m/min) and increments of 1 m/min) every minute were applied until 18 m/min for a maximum running time of 60 min.

### Statistical Analysis

Results are presented as means ± standard error of the mean (SEM) or, when applicable, means ± SEM with individual data points shown. Statistical analyses were performed using unpaired *t*-test to compare muscles from two different experimental conditions. Interactions between FS electroporation or sActRIIB treatment and *Pak1* deletion were assessed using a two-way ANOVA followed by Bonferroni post-tests. Fiber CSA distribution statistical analysis was performed using χ^2^ Pearson test. Statistical significance was set at *P-value* < 0.05.

## Results

### Global Comparative Transcriptomic Analysis

To decipher the molecular mechanisms behind the muscle phenotype induced by MSTN inhibition, we sought to compare eight published transcriptomic analyses encompassing various models of MSTN inhibition: three models of MSTN gene deletion, whose two prenatal (Steelman et al., 2006; Rahimov et al., 2011) and one postnatal (Welle et al., 2009); two models of postnatal MSTN inhibition with either acute (sActRIIB 1x) or chronic (sActRIIB 4x) sActRIIB treatment (Rahimov et al., 2011); three models of FS-induced MSTN inhibition, whose two postnatal with either acute (72 h post-transfection) or chronic (7–14 days post-transfection) FS electroporation (Davey et al., 2016) and one prenatal with muscle–specific transgenic mTrFS mice (Barbé et al., 2017). Prior to the comparison, the microarray data were uploaded for chip matching and official gene symbols. Only differentially expressed genes with *P-value* < 0.05 were integrated in the comparative analysis, except for data published by Welle et al. (2009), for which a *P-value* < 0.01 was applied. With a 1.5-fold cut-off, 135 genes were commonly regulated in at least one of each MSTN inhibition model, namely MSTN deletion, sActRIIB treatment, and FS overexpression. Differential expression of 20 genes (8 upregulated and 12 downregulated) was shared between at least 6 from the 8 transcriptomes compared (Table 2).

**TABLE 2:** Commonly regulated genes following MSTN inhibition.

	MSTN			FS			sActRIIB	
Genes	KO	KO	KO	mTrFS	Acute	Chronic	Acute	Chronic
	Steelman et al. (2006)	Rahimov et al. (2011)	Welle et al. (2009)	Barbé et al. (2017)	Davey et al. (2016)	Davey et al. (2016)	Rahimov et al. (2011)	Rahimov et al. (2011)
	FC	FC	FC	FC	FC	FC	FC	FC
*Pak1*	2.1	1.7	1.6	2.0	1.8	2.9	1.5	1.7
*Gdap1*	1.6	1.6	1.9	2.3	1.8	1.7	1.4	**X**
*Igfbp5*	**X**	1.9	1.6	1.6	1.5	1.6	1.7	1.7
*Mybph*	2.9	1.6	**X**	2.5	3.3	10.6	1.6	1.6
*Xrcc5*	1.8	1.5	1.5	1.9	1.4	1.7	**X**	1.4
*Inmt*	−2.1	−2.9	**X**	−3.1	−1.8	−4.2	−2.7	−2.4
*Nos1*	**X**	−1.4	−1.4	−1.7	−2.2	−3.8	−2.2	−1.6
*Lmod2*	−2.1	−2.9	**X**	−2.5	−2.0	−3.2	−1.8	−2.0
*Ddah1*	−2.8	−1.5	−2.1	**X**	−3.3	−5.4	−1.6	−1.4
*Ramp1*	−1.5	−1.9	−2.1	−1.7	−1.6	−2.6	**X**	−1.4
*A930003A15Rik*	2,4	1,5	**X**	**X**	3,2	15,7	1,8	2,0
*Hspb8*	1.8	1.5	**X**	2.1	1.6	3.4	**X**	1.4
*Atp1b4*	2.2	**X**	**X**	1.8	1.7	1.7	1.7	1.5
*Tbc1d1*	−2.7	−1.5	**X**	−2.2	−2.2	−3.4	−2.3	**X**
*Asb15*	−1.6	−1.9	**X**	**X**	−1.6	−3.4	−1.9	−1.4
*Itgb5*	**X**	−1.8	**X**	−2.0	−1.9	−2.6	−1.6	−1.4
*Skil*	−1.6	**X**	−1.4	−2.3	−2.2	−3.2	−1.4	**X**
*Ptpn3*	−2.3	−3.1	**X**	**X**	−1.7	−2.7	−1.4	−2.0
*Dusp26*	**X**	−1.8	−1.6	−2.1	−1.5	−2.3	**X**	−1.6
*Fxyd6*	−2.3	−1.5	−1.6	−1.9	**X**	−2.0	**X**	−1.4

*FC, Fold-change compared to CTRL condition; X, not found; *Pak1*, p21-activated kinase 1; *Gdap1*, Ganglioside-induced differentiation-associated-protein 1; *Igfbp5*, Insulin-like growth factor binding protein 5; *Mybph*, Myosin binding protein H; *Xrcc5*, X-ray repair complementing defective repair in Chinese hamster cells 5; *Inmt*, Indolethylamine N-methyltransferase; *Nos1*, Nitric oxide synthase 1; *Lmod2*, Leiomodin 2; *Ddah1*, Dimethylarginine dimethylaminohydrolase 1; *Ramp1*, Receptor (calcitonin) activity modifying protein 1; *Hspb8*, Heat shock protein 8; *Atp1b4*, ATPase, (Na +)/K + transporting, beta 4 polypeptide; *Tbc1d1*, TBC1 domain family, member 1; *Asb15*, Ankyrin repeat and SOCS box-containing 15; *Itgb5*, Integrin beta 5; *Skil*, SKI-like; *Ptpn3*, Protein tyrosine phosphatase, non-receptor type 3; *Dusp26*, Dual specificity phosphatase 26; *Fxyd6*, FXYD domain-containing ion transport regulator 6.*

Then, we investigated the biologic processes coordinated by these 20 differentially expressed genes according to information extracted from Database for Annotation, Visualization and Integrated Discovery (DAVID)^1^ and NCBI^2^. Consistent with the molecular mechanisms responsible for the muscle hypertrophy induced by MSTN inhibition, several genes were associated to Insulin/IGF-I and Smad signaling pathways (Table 3). Among the three genes associated to Insulin/IGF-I signaling (*Pak1*, *Igfbp5*, and *Tbc1d1*), *Pak1* was the only gene consistently upregulated in all MSTN inhibition models compared in Table 2. Given the role of *Pak1* in muscle mass regulation (Martinelli et al., 2016; Joseph et al., 2017; Cerquone Perpetuini et al., 2018), we investigated its action in the muscle mass increase induced by MSTN inhibition.

**TABLE 3:** Biological processes regulated by the commonly regulated genes following MSTN inhibition.

Biological Process	Genes

Signal transduction	
Insulin/IGF-I signaling	*Pak1, Igfbp5, Tbc1d1*
Smad signaling	*Skil*
EGFR signaling	*Ptpn3*
MAPK signaling	*Dusp26*
CGRP signaling	*Ramp1*
NO-mediated signaling	*Nos1*

**Cell organization**	

Mitochondrial organization	*Gdap1*
Myofilaments and Cytoskeleton	*Mybph, Lmod2, Itgb5*

**Proteostasis**	

Proteosynthesis	*Xrcc5, Atp1b4*
Protein folding	*Hspb8*
Proteolysis	*Asb15*

**Detoxification and Cytoprotection**	

Detoxification of selenium compounds	*Inmt*
Regulation of NO generation	*Ddah1*

**Transport**	

Sodium ion transport	*Fxyd6*

*EGFR, Epidermal growth factor receptor; MAPK, Mitogen-activated protein kinase; CGRP, Calcitonin gene-related peptide; NO, Nitric oxide.*

### Muscle Hypertrophy Induced by MSTN Inhibition Is Associated With Increased *Pak1* Levels

**FIGURE 1:**
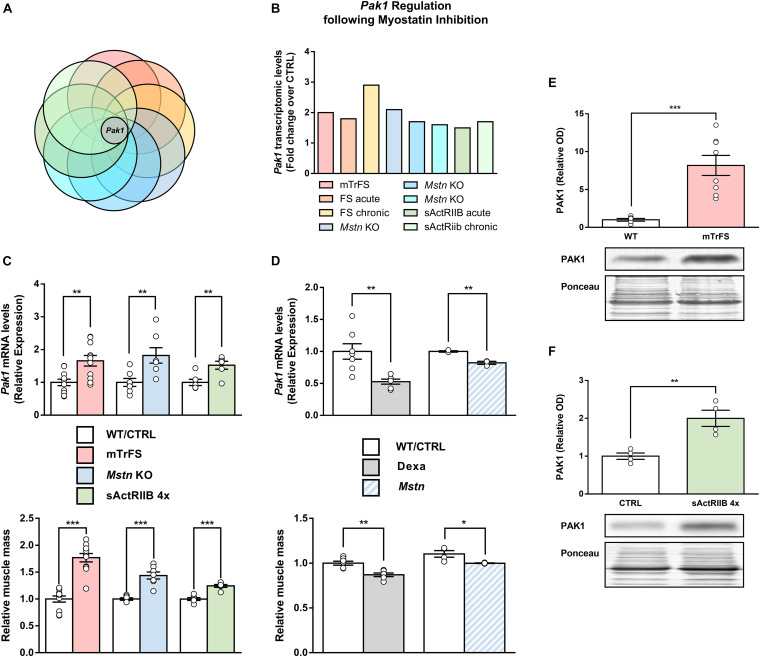
Global comparative transcriptomic analysis of skeletal muscles from mouse models of MSTN inhibition identified *Pak1* as a MSTN-responsive gene. **(A)** Venn diagram comparing the differentially expressed genes into muscles from eight different models of pre- and postnatal MSTN inhibition with *Pak1* as the only gene commonly regulated between all the transcriptomes compared. **(B)** *Pak1* mRNA fold-changes for each muscle transcriptome studied. **(C)** Muscle *Pak1* mRNA levels and muscle weight from TA muscles of mTrFS vs. WT mice (*n* = 11/group); from TA muscles of four times sActRIIB-injected vs. PBS-injected mice (*n* = 6/group) and from GC muscles of MSTN KO vs. WT mice (*n* = 7/group). **(D)** Muscle *Pak1* mRNA levels and muscle weight from GC muscles of DEXA-treated vs. saline-treated mice (*n* = 7/group) and from MSTN-transfected vs. CTRL pcDNA-transfected TA muscles (*n* = 3/group). Representative western blot image and densitometric analysis of PAK1 protein levels in GC muscles from **(E)** mTrFS vs. WT C57Bl/6 mice (*n* = 8/group) and from **(F)** sActRIIB-injected vs. PBS-injected mice (*n* = 4/group). Results are expressed as means ± SEM and statistical analysis was performed using unpaired *t*-test (*P < 0.05; **P < 0.01, and ***P < 0.001 vs. CTRL/WT).

As illustrated in Figures 1A,B, *Pak1* was the only gene consistently upregulated in all MSTN inhibition models. To confirm the validity of these results, the muscle mRNA levels of *Pak1* were assessed by RT-qPCR in several models of MSTN inhibition. As expected, muscle *Pak1* expression was systematically increased in skeletal muscles from mTrFS (+66%, *P* < 0.01), MSTN KO (+82%, *P* < 0.01) and 4 times sActRIIB-injected mice (+52%, *P* < 0.01) compared to WT or CTRL mice, as was muscle mass (mTrFS: +77%, *P* < 0.001; MSTN KO: +53%, *P* < 0.001; and sActRIIB: +25%, *P* < 0.001) (Figure 1C). In contrast, muscle *Pak1* expression was decreased in atrophied muscles following MSTN transfection (*Pak1* mRNA: −18%, *P* < 0.01; muscle mass: −9%, *P* < 0.05), compared to CTRL pcDNA-transfected muscles (Figure 1D). Moreover, mice treated with DEXA, a well-known atrophic agent acting in part through the induction of MSTN (Schakman et al., 2013), also showed a similar decrease in *Pak1* mRNA and muscle mass (*Pak1* mRNA: −46%, *P* < 0.05; muscle mass: −13%, *P* < 0.01), compared to their control littermates (Figure 1D). Finally, as shown in Figures 1E,F, overexpression of FS and sActRIIB treatment increased not only *Pak1* mRNA but also its protein expression in skeletal muscle (mTrFS: 8.2 ± 1.3-fold vs. WT, *P* < 0.001; sActRIIB: 2.0 ± 0.2-fold vs. CTRL, *P* < 0.01). Altogether, these results confirm that muscle PAK1 levels are consistently upregulated in response to MSTN inhibition, suggesting PAK1 could contribute to driving the hypertrophic muscle phenotype induced by MSTN inhibition.

### Deletion of *Pak1* Does Not Alter Muscle Mass and Function

**FIGURE 2:**
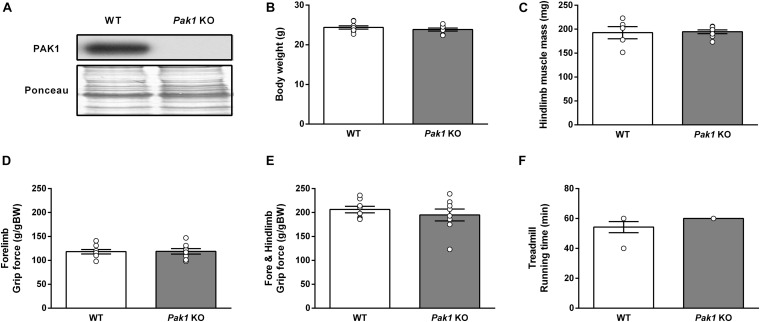
Deletion of *Pak1* does not alter muscle mass and function. **(A)** Representative western blot image of muscle PAK1 protein levels in GC muscles from *Pak1* KO vs. WT mice. **(B)** Body weight, and **(C)** hindlimb muscle mass (including TA, GC, SOL, and EDL muscles) from *Pak1* KO vs. WT mice (*n* = 5–8/group). **(D,E)** Muscle strength from *Pak1* KO vs. WT mice (*n* = 7–8/group). Mice were lowered on a grid connected to a sensor to measure the muscle force of their forelimbs or of both their forelimb and hindlimb and data were then expressed in gram force relative to body weight (g/gBW). **(F)** Endurance tolerance from *Pak1* KO vs. WT mice (*n* = 7–8/group). Mice were submitted to an incremental treadmill running exercise (from 5 m/min with added 1 m/min every min until maximum 18 m/min) for a maximum running time of 60 min. Results are expressed as means ± SEM and statistical analysis was performed using unpaired *t*-test to compare results from *Pak1* KO and WT mice.

To investigate the role of PAK1 in mediating the MSTN inhibition effect on muscle mass and function, we investigated the consequences of *Pak1* deletion on muscle homeostasis. The absence of *Pak1* in skeletal muscle from *Pak1* KO mice was confirmed by western blot (Figure 2A). There was no difference in body weight nor muscle mass between *Pak1* KO and WT mice (Figures 2B,C). Two different functional tests were used to investigate the global muscular force (grip test) and the resistance to fatigue (treadmill running test) which were unchanged under *Pak1* deletion (Figures 2D–F).

### Role of *Pak1* in the Magnitude of Muscle Hypertrophy Induced by MSTN Inhibition

**FIGURE 3:**
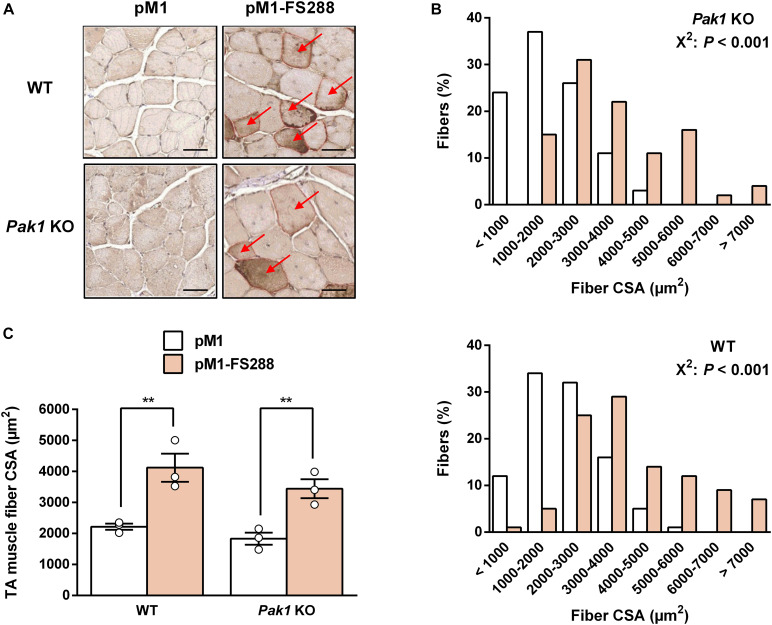
Follistatin induces skeletal muscle hypertrophy despite the absence of *Pak1*. **(A)** Representative FS-c-myc immunochemistry image from TA muscle of *Pak1* KO and WT littermate mice. Red arrows indicate C-myc positive fibers. Scale bar = 50 μm. **(B)** Muscle fiber size distribution from pM1-FS288-transfected vs. pM1-transfected TA muscles of *Pak1* KO (up panel) and WT (bottom panel) mice (*n* = 3/group). **(C)** Muscle fiber CSA assessed 17 days after electroporation of pM1-hFS288 (orange columns) or pM1 (white columns) in TA muscles from *Pak1* KO vs. WT littermate mice (*n* = 3/group). Results are expressed as mean ± SEM. Statistical analysis was performed using two-way ANOVA test and Bonferroni post-tests (FS effect: **P < 0.01). Fiber CSA distribution statistical analysis was performed using χ^2^ Pearson test (*P* < 0.001).

Given the role of *Pak1* in muscle mass regulation (Joseph et al., 2017; Cerquone Perpetuini et al., 2018) and its increased muscle levels in response to MSTN inhibition, we investigated the effect of *Pak1* deletion on muscle hypertrophy induced by FS electroporation. For this purpose, we overexpressed FS by DNA electrotransfer in TA muscle from *Pak1* KO mice. Surprisingly, the absence of *Pak1* did not affect the FS-induced skeletal muscle hypertrophy. Indeed, we observed a similar increase in muscle fiber CSA from *Pak1* KO (+96%, 3.443 ± 306 vs. 1.831 ± 194 μm^2^, *P* < 0.01) and WT mice (+85%, 4.121 ± 452 vs. 2.218 ± 101 μm^2^, *P* < 0.01) in response to FS overexpression (Figures 3A,C). These results were supported by the analysis of fiber size distribution showing a similar shift to a greater proportion of large fibers among the FS-transfected than among CTRL fibers in the two groups of mice (*P* < 0.001, Figure 3B). These results demonstrate that FS retains its hypertrophic effect toward skeletal muscle despite the absence of *Pak1*.

### Role of *Pak1* in the Onset of Muscle Hypertrophy Induced by MSTN Inhibition

**FIGURE 4:**
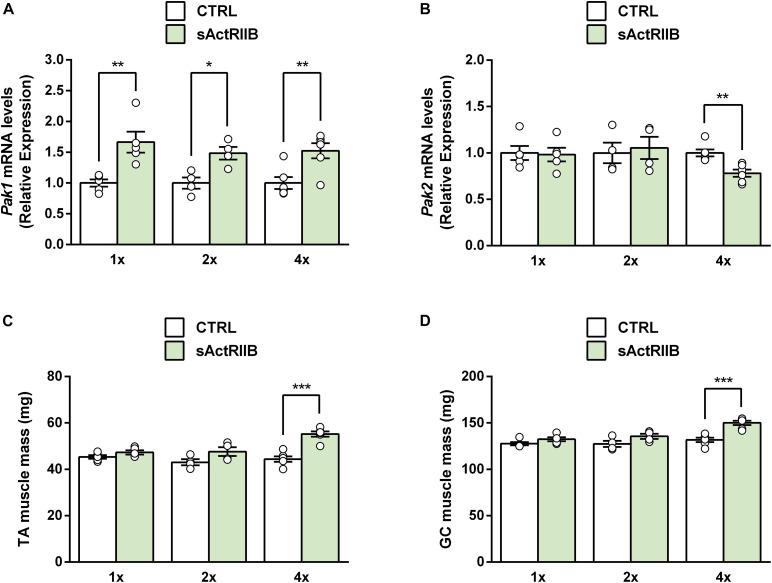
Muscle *Pak1* and *Pak2* mRNA levels in response to skeletal muscle hypertrophy induced by MSTN inhibition. **(A)** TA muscle *Pak1* and **(B)** *Pak2* mRNA levels, **(C)** TA and **(D)** GC muscle weight after one, 2 or 4 injections of sActRIIB (green columns) or PBS (white columns) in WT mice (*n* = 4–6/group). Results are expressed as means ± SEM. Statistical analysis was performed using unpaired *t*-test to compare results from sActRIIB-treated and PBS-treated mice (*P < 0.05; **P < 0.01, and ***P < 0.001 vs. CTRL).

Although *Pak1* deletion does not change the extent of muscle hypertrophy induced by FS overexpression, we could not rule out that *Pak1* does not regulate the onset of skeletal muscle hypertrophy. Indeed, the transcriptomic data from acute models of MSTN inhibition (sActRIIB 1x and FS acute; Figure 1B) show an acute upregulation of *Pak1* suggesting that its increase may not result from muscle mass increase but could be involved in its onset. Previous results demonstrated that 4 injections of sActRIIB induces a maximal hypertrophy (Hoogaars et al., 2012; Hulmi et al., 2013). However, we observed that muscle *Pak1* mRNA was already upregulated after one (+64%, *P* < 0.01) or 2 (+49%, *P* < 0.05) injections of sActRIIB compared to PBS-injected mice (Figure 4A), while muscle hypertrophy only appears after 4 injections (Figures 4C,D). For this reason, we investigated the effect of *Pak1* deletion at the start and the end of muscle hypertrophy process induced by sActRIIB treatment, namely after 2 and 4 injections, respectively.

**FIGURE 5:**
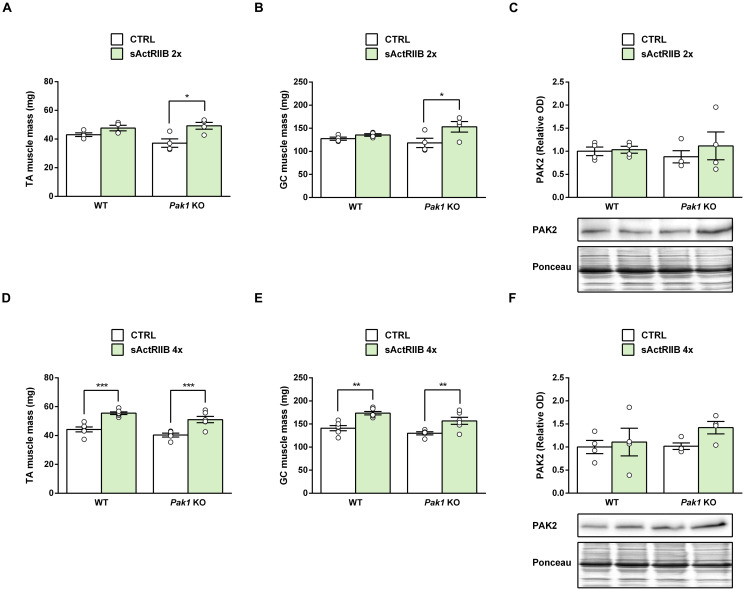
sActRIIB induces skeletal muscle hypertrophy despite the absence of *Pak1*. **(A,D)** TA and **(B,E)** GC muscle weight and **(C,F)** TA muscle PAK2 protein levels after 2 injections (*n* = 4/group) or 4 injections (*n* = 4–6/group) of sActRIIB (green columns) or PBS (white columns) in *Pak1* KO vs. WT mice. Results are expressed as means ± SEM. Statistical analysis was performed using two-way ANOVA test and Bonferroni post-tests (sActRIIB effect: *P < 0.05; **P < 0.01, and ***P < 0.001).

In response to 2 injections of sActRIIB, while skeletal muscle hypertrophy was only starting in WT mice (TA muscle: +11%, 48 ± 2 vs. 43 ± 1 mg, *ns;* GC muscle: +6%, 136 ± 3 vs. 127 ± 3 mg, *ns*), we observed a significant increase in muscle weight from *Pak1* KO (TA muscle: +33%, 49 ± 2 vs. 37 ± 3 mg, *P* < 0.05; GC muscle: +29%, 153 ± 11 vs. 118 ± 10 mg, *P* < 0.05) (Figures 5A,B). However, in response to 4 injections of sActRIIB, the maximal magnitude of skeletal muscle hypertrophy was similar in *Pak1* KO (TA muscle: +26%, 51 ± 2 vs. 40 ± 1 mg, *P* < 0.001; GC muscle: +21%, 157 ± 8 vs. 130 ± 3 mg, *P* < 0.01) and WT mice (TA muscle: +25%, 56 ± 1 vs. 44 ± 2 mg, *P* < 0.001; GC muscle: +23%, 173 ± 4 vs. 141 ± 3 mg, *P* < 0.01) (Figures 5D,E). These results confirm that *Pak1* deletion does not impede the skeletal muscle hypertrophy, regardless of the MSTN inhibition model.

Because PAK1 and PAK2 seem to be redundant, we hypothesized that *Pak1* deletion could be compensated by *Pak2* upregulation. However, we did not observed any significant change in TA muscle PAK2 protein levels following *Pak1* deletion (Figures 5C,F). In response to sActRIIB, we observed that TA muscle *Pak2* mRNA was downregulated only after 4 injections of sActRIIB (−23%, *P* < 0.01) compared to PBS-injected mice (Figure 4B), once the skeletal muscle mass increased (Figures 4C,D). However, we did not observe any significant change in muscle PAK2 protein levels of *Pak1* KO and WT mice in response to 2 or 4 sActRIIB-injections (Figures 5C,F).

### Molecular Mechanisms Underlying sActRIIB-Induced Muscle Growth in *Pak1* KO Mice

**FIGURE 6:**
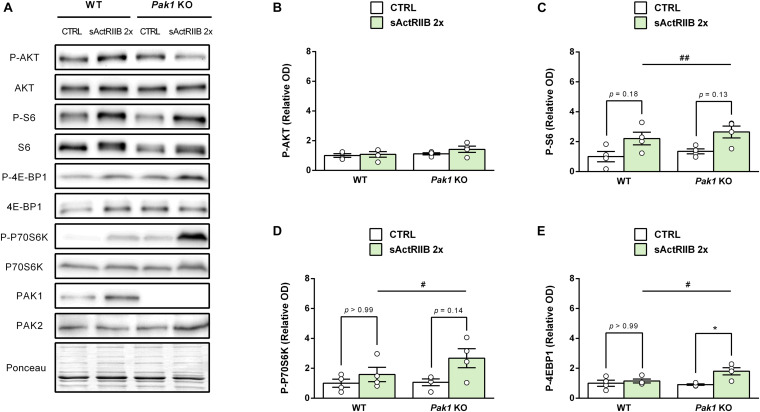
*Pak1* deletion tends to increase the sActRIIB-induced 4E-BP1 inactivating phosphorylation. **(A)** Representative western blot images and densitometric analysis of **(B)** P-AKT, **(C)** P-S6, **(D)** P-P70S6K, and **(E)** P-4E-BP1 levels in GC muscles from *Pak1* KO and WT mice after 2 injections of sActRIIB (green columns) or PBS (white columns) (*n* = 4/group). The results are expressed as mean ± SEM. Statistical analysis was performed using two-way ANOVA tests (sActRIIB effect: ^#^*P* < 0.05 and ^##^*P* < 0.01) and Bonferroni post-tests (sActRIIB effect: *P < 0.05).

**FIGURE 7:**
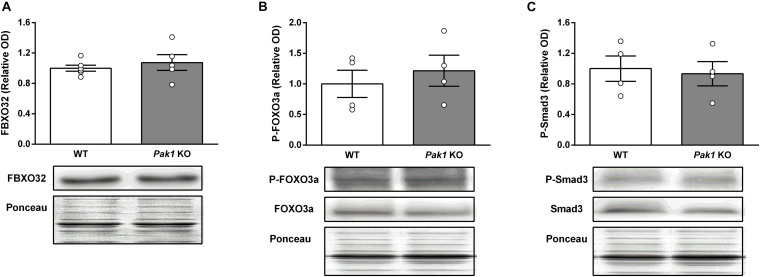
*Pak1* deletion does not increase Smad3 phosphorylation nor FBXO32 protein expression in skeletal muscle. **(A)** GC muscle FBXO32 protein levels, **(B)** P-FOXO3a, and **(C)** P-Smad3 levels in GC muscles from *Pak1* KO and WT mice (*n* = 4–6/group). The results are expressed as mean ± SEM. Statistical analysis was performed using unpaired *t*-test to compare results from *Pak1* KO and WT mice.

To gain new insights on the molecular mechanisms responsible for the observed changes in muscles from *Pak1* KO mice after 2 sActRIIB-injections, we investigated proteins involved in muscle growth. In line with previous data (Hulmi et al., 2013), the phosphorylation state of AKT at Ser^473^ was unchanged (Figures 6A,B). In contrast, we observed an increased phosphorylation of S6 ribosomal protein at Ser^235/236^ (*Pak1* KO mice: 2.3 ± 0.6-fold; WT mice: 2.2 ± 0.4-fold; *P* < 0.01, *n* = 4/group; Figures 6A,C), indicating activated mTORC1 signaling. Interestingly, phosphorylation of 4E-BP1 at Ser^65^ and P70S6K at Thr^389^ seemed to be more increased by sActRIIB in *Pak1* KO (P-4E-BP1: 1.6 ± 0.3-fold, *P* < 0.05; P-P70S6K: 2.7 ± 0.6-fold, *P* = 0.14; *n* = 4/group) than in WT mice (P-4E-BP1: 1.2 ± 0.1-fold, *ns*; P-P70S6K: 1.6 ± 0.5-fold, *ns*; *n* = 4/group) (Figures 6A,D,E). Although the small number of samples creates low statistical power, our results suggest that *Pak1* deletion could increase the rate of translation initiation through activating phosphorylation of P70S6K and inactivating phosphorylation of 4E-BP1. Since Smad3 and FBXO32 emerged as the downstream effectors in the anti-hypertrophic action of *Pak1* in the heart (Liu et al., 2011; Tsui et al., 2015), we investigated these signaling proteins in skeletal muscle from *Pak1* KO mice. However, we did not observe any change in FBXO32 protein expression, nor in P-FOXO3a or P-Smad3 levels in *Pak1* KO muscles (Figure 7).

## Discussion

By comparing changes in muscle transcriptome associated with muscle hypertrophy induced by various MSTN inhibition approaches, we identified *Pak1* as the only gene systematically upregulated regardless the MSTN inhibition model considered. This *in silico* observation was confirmed by RT-qPCR analysis: muscle *Pak1* mRNA levels were increased in parallel to skeletal muscle hypertrophy in mTrFS, MSTN KO, and sActRIIB-treated mice. At the opposite, we observed a decreased expression of muscle *Pak1* mRNA levels in atrophied muscles following MSTN transfection and DEXA treatment. Using *Pak1* KO mice, we investigated the effect of *Pak1* deletion on muscle hypertrophy induced by FS electroporation and sActRIIB treatment. Although we observed a slight acceleration in the sActRIIB-induced muscle hypertrophy, our results show that FS and sActRIIB retain their anabolic effect toward skeletal muscle even in the absence of *Pak1*. Taken together, our results demonstrate that *Pak1* is dispensable for the muscle mass increase caused by MSTN inhibition.

Several studies examined the transcriptomic changes caused by MSTN deletion during pre- (Steelman et al., 2006; Chelh et al., 2009; Rahimov et al., 2011) and post-development (Welle et al., 2009) or in response to pre- (Barbé et al., 2017) and postnatal MSTN inhibition (Rahimov et al., 2011; Miao et al., 2015; Davey et al., 2016). Due to the extremely large number of data resulting from these high-throughput experimental techniques, interpretation of these analysis is made difficult. Although we penned only one of these transcriptomic analyses, our work combines in a unique way all the results obtained from various models of MSTN inhibition and offers a short list of 20 genes (8 upregulated and 12 downregulated) whose mRNA levels appear commonly regulated in most (75%) MSTN inhibition models compared. In this study, we highlighted *Pak1* as a likely mediator of the skeletal muscle hypertrophy induced by MSTN inhibition based on its consistent induction in all MSTN inhibition models. The fact that *Pak1* is also induced by the Insulin/IGF-I pathway (Tunduguru et al., 2014), which contributes to the hypertrophy induced by the inhibition of MSTN (Kalista et al., 2012; Barbé et al., 2015), supports this hypothesis. The role of the 19 other regulated genes remains to be investigated.

p21-activated kinase 1 is the best characterized member of the PAK kinases and has been involved in actin cytoskeleton remodeling (Sells et al., 1997). In recent years, a role for *Pak1* in muscle mass regulation has emerged, notwithstanding some inconsistencies. *Pak1* has been first suggested to contribute to the regulation of cardiac muscle hypertrophy. Indeed, while some authors found that *Pak1* is essential for AKT activity in cardiomyocytes (Mao et al., 2008), others reported an anti-hypertrophic action of *Pak1* in the heart (Liu et al., 2011). Regarding the skeletal muscle, Joseph et al. (2017) demonstrated that muscle-specific combined *Pak1* and *Pak2* deletion resulted in reduced skeletal muscle mass. Moreover, a decreased expression of *Pak1* was observed in cancer-associated cachectic muscles from Yoshida hepatoma-bearing rats (Martinelli et al., 2016) and colon adenocarcinoma C26-bearing mice (Cerquone Perpetuini et al., 2018). Most of all, *Pak1* overexpression prevented the muscle atrophy observed in cachectic muscles suggesting that *Pak1* might have an anti-atrophic action in skeletal muscle (Cerquone Perpetuini et al., 2018). Reasoning that PAK1 has a central role in skeletal muscle mass regulation and its muscle levels increase in response to MSTN inhibition, we hypothesized that PAK1 could mediate the skeletal muscle hypertrophy induced by MSTN inhibition. Although this assumption was never been investigated, it was suggested that the anti-hypertrophic action of PAK1 in cardiac muscle implicates Smad3 (Tsui et al., 2015), the main downstream effector of the atrophic effect of MSTN (Sartori et al., 2009). Moreover, it has been evidenced that FS overexpression amplifies insulin-stimulated phosphorylation of PAK1 at its activation site Thr^423^ (Han et al., 2019), while our previous data have demonstrated the role of Insulin signaling in the FS-induced skeletal muscle hypertrophy (Kalista et al., 2012; Barbé et al., 2015). For these reasons, we investigated the effect of *Pak1* deletion on skeletal muscle hypertrophy induced by MSTN inhibition.

In the present work, we show that *Pak1* deletion does not alter skeletal muscle mass, just as ablation of *Pak1* has no effect on muscle fiber size (Joseph et al., 2017), suggesting that, in basal conditions, changes in *Pak1* expression levels are not sufficient to affect muscle mass. In contrast, in pathological conditions, deletion of *Pak1* is sufficient to amplify cardiac muscle hypertrophy in response to pressure overload (Liu et al., 2011), while *Pak1* overexpression mitigates muscle atrophy caused by cancer cachexia (Cerquone Perpetuini et al., 2018). In addition, we demonstrate that *Pak1* deletion does not affect skeletal muscle function. The absence of consequences of *Pak1* deletion on skeletal muscle mass and function is striking, giving the many roles of this protein in particular on the actin cytoskeleton. Since we used a developmental KO model, compensation for the lack of *Pak1* is still possible. In particular, *Pak1* and *Pak2* have been shown to be redundant regulators of postnatal myogenesis (Joseph et al., 2017). Therefore, we may not exclude that *Pak2* compensates for the lack of *Pak1*. However, the absence of changes in TA muscle PAK2 protein levels observed in *Pak1* KO mice does not support this hypothesis.

An interesting finding in the current study is that *Pak1* is induced prior to the muscle mass increase suggesting that increased *Pak1* levels do not result from muscle hypertrophy but from MSTN inhibition itself and may modulate the rate of the anabolic process. Indeed, our results showed that 2 sActRIIB injections were sufficient to induce muscle hypertrophy in *Pak1* KO mice, while 4 injections are needed to increase muscle mass at the same extent in WT mice. These results suggest a negative role for *Pak1* in the rate of muscle hypertrophy process. Although we did not measure the muscle fiber CSA, the sActRIIB is well known to increase muscle fiber CSA in parallel to muscle mass, without affecting the number of muscle fibers (Cadena et al., 2010; Pistilli et al., 2010; Hulmi et al., 2013; Relizani et al., 2014). After 2 sActRIIB injections, we observed a slight increase in phosphorylated 4E-BP1 and P70SK in *Pak1* KO mice suggesting that *Pak1* could curb muscle hypertrophy through inhibiting these protein targets. Supporting this hypothesis, Smad3, and FBXO32 has emerged as the downstream effectors in the anti-hypertrophic action of *Pak1* in the heart (Liu et al., 2011; Tsui et al., 2015). However, we did not observe any change in Smad3 phosphorylation nor FBXO32 protein expression in skeletal muscle from *Pak1* KO mice. If these results are due to genetic compensation for the lack of *Pak1* or if *Pak1* does not signal through Smad3 and FBXO32 in skeletal muscle remains unknown. Therefore, although these findings may seem appealing, more research is definitely needed before defending this thesis, given that our sample size was low and that muscle fiber CSA was not closely assessed in this experiment.

The major discovery in the current study is that *Pak1* is dispensable for the skeletal muscle hypertrophy induced by MSTN inhibition. Due to the plateau effect occurring with the sActRIIB injected four times at 10 mg/kg (Hoogaars et al., 2012; Hulmi et al., 2013), we cannot exclude *Pak1* deletion would not affect the magnitude of muscle hypertrophy induced by the sActRIIB. However, the similar results observed in response to FS overexpression suggest that muscle hypertrophy induced by MSTN inhibition does not rely on *Pak1*. Moreover, the dispensable nature of *Pak1* for the muscle hypertrophy caused by MSTN inhibition is in line with the recent study of Møller et al. (2020) who have demonstrated that muscle-specific genetic ablation of *Pak2*, but not the whole-body *Pak1* knockout, impairs the insulin-induced muscle glucose uptake, the second main effect of MSTN inhibition toward muscle (Akpan et al., 2009; Guo et al., 2009; Morissette et al., 2009; Cleasby et al., 2014). In our work, we observed that, in response to sActRIIB, the muscle *Pak2* mRNA levels were decreased only once the muscle mass increased. Knowing that combined deletion of *Pak1* and *Pak2* reduces muscle mass (Joseph et al., 2017), the decreased *Pak2* levels might result from muscle hypertrophy and acts as a brake for the anabolic signal. Alternatively, given the role of *Pak2* in the insulin-stimulated glucose uptake in skeletal muscle, we might assume that changes observed in muscle *Pak2* mRNA levels would curb the increased insulin sensitivity induced by the sActRIIB as a negative feedback. However, we did not observe any significant change in PAK2 protein levels from *Pak1* KO and WT mice treated with sActRIIB.

To the best of our knowledge, we are the first to have investigated the role of *Pak1* in the skeletal muscle hypertrophy induced by MSTN inhibition. The strength of our observations stems in part from the use of different MSTN inhibition models. Moreover, the sActRIIB seems to have clinical relevance as therapeutic strategy for reversing muscle wasting, such as sarcopenia and cachexia, as well as muscular dystrophies (Saitoh et al., 2017). In this regard, defining the key mediators of MSTN actions on skeletal muscle mass is essential to prevent and to remedy the potential adverse effects of investigational drugs. In this regard, our work demonstrates that, *Pak1* is permissive for the anabolic action induced by MSTN inhibition. Further studies will need to investigate more deeply the role of group I PAKs in the skeletal muscle hypertrophy induced by MSTN inhibitors.

## Data Availability Statement

The original contributions presented in the study are included in the article/supplementary material, further inquiries can be directed to the corresponding author.

## Ethics Statement

The animal study was reviewed and approved by Committee for Ethical Practices in Animal Experiments of the Catholic University of Louvain (Brussels, Belgium).

## Author Contributions

CB, AL, OR, and J-PT conceived and planned the experiments. CB, AL, and PL carried out the experiments. CB, AL, PL, and J-PT analyzed and interpreted the data. CB wrote the manuscript with support from J-PT. All authors provided critical feedback and helped shape the research, analysis, and manuscript.

## Conflict of Interest

The authors declare that the research was conducted in the absence of any commercial or financial relationships that could be construed as a potential conflict of interest.

## Footnotes

1 http://david.abcc.ncifcrf.gov

2 https://www.ncbi.nlm.nih.gov/
